# Address, Introductory to the Annual Course of Lectures in Rush Medical College

**Published:** 1857-12

**Authors:** N. S. Davis


### THE NORTH-WESTERN

### MEDICAL AND SURGICAL JOURNAL.

(NEW SERIES.)

VOL. VI. DECEMBER, 1857. NO. 12.

### ORIGINAL COMMUNICATIONS.

### ARTICLE I.

### ADDRESS, INTRODUCTORY TO THE FIFTEENTH ANNUAL COURSE OF LECTURES IN RUSH MEDICAL COLLEGE;

DELIVERED NOVEMBER 2, 1857.

BY N. S. DAVIS, M. D.

Gentlemen,—It is with pleasure that I rise, and in behalf of my colleagues and myself, extend to you a cordial welcome to the halls of this College, and thus formally inaugurate another annual course of instruction upon those varied and important topics which constitute the science and art of medicine. To the thoughtful and intelligent mind, some of these topics possess an interest scarcely equaled in any other department of human learning; while the application of the truths embraced in them all, to the great work of preventing, alleviating and curing disease, constitutes the noblest of human employments. It is no part of my present purpose, however, to eulogize either the science or the practice of our profession. Neither shall I attempt to cull from the pages of its ample and varied literature, a few of the brighter gems with which to beguile the passing hour. On the contrary, I shall invite your attention to a topic which is intimately connected with the further prosecution of your studies; and a careful consideration of which may be of much service to some of you hereafter. Every student should have two things clearly impressed upon his mind, namely, first, the special topics or branches of learning necessary for him to investigate, and, second, the various methods by which the investigation can be conducted to the most successful and valuable results. It is to the last named topic that I shall chiefly direct your attention on the present occasion.

Medical investigators have developed various methods of research during the past history of our art; and though since the promulgation of the Baconion philosophy, all the methods claim to be founded on the principles of induction, yet at no period of time has there been discernible a greater variety than during the last half century. Whatever may be the apparent diversity, however, they may all be appropriately arranged under three heads, namely, those dependent on simple observation; those conducted chiefly by reflection, and often styled rational or theoretical methods; and those based upon experiment. The first is the most ancient, and numbers among its disciples some of the most illustrious men whose names adorn the pages of history. Perhaps none have ever attained a higher reputation in the school of observation, or presented an example of patient industry, minuteness of attention, and candor, more worthy of imitation than Hippocrates himself. Through most of the long period extending from the time of Hippocrates to the middle of the eighteenth century, medical investigations partook much more of the nature of theoretical dogmas than of observed facts. From the latter period to the close of that century, all the various branches of natural science became rapidly developed, and their intimate relations to medicine became more apparent at every step. Analytical chemistry began to shed an enduring ray of light upon the composition and qualities of many of the secretions and structures of the human body; thereby not merely disproving former vague and theoretical doctrines, but adding immensely to the number of demonstrated facts in the interesting departments of physiology and pathology. An application of the same department of chemistry to investigations concerning the nature and composition of medicinal agents soon effected improvements in the Materia Medica of the most gratifying character. Applications of principles and laws derived from other branches of natural science were made during the early part of the present century of no less importance than the preceding. Such was the application of acoustics, or the laws of transmission of sound, to the diagnosis of disease by Læennec and Piorry, in the now familiar acts of auscultation and percussion.

As it was by a rigid observation of facts, aided now and then by well-devised experiments, that the different branches of natural science were so rapidly transformed from a crude jumble of facts and fanciful theories to the form of well-defined and exact sciences, so the many points of contact between these sciences and the several branches of medicine could not fail to render the same method of investigation popular and predominant in the profession.

Hence, it soon became popular among medical teachers and writers to declaim against all attempts to theorize, or establish *systems* in medicine based on a few alleged fundamental laws, as had been done by Brown, Darwin, Cullen, Broussais, Rush, and others.

One well observed fact was declared to be worth an hundred theories. The consequence is that in this our day, the simple observation and classification of facts constitutes the predominating and popular method of advancing both the science and the art of our profession. In this widely extended school of observation may be traced several subordinate divisions, each worthy of a passing notice. The first, following the example of Sydenham, fix the attention upon the phenomena of disease as presented at the bed-side, and upon the actual results of treatment in each individual case. The great object with the members of this division is to become familiar with the circumstances and causes which are capable of originating or modifying disease, with the essential phenomena and tendencies of each form of morbid action, and with the effects of remedial agents in mitigating or curing the same.

This may be appropriately styled the *clinical method* of observation; and amongst its followers must be ranked far the larger portion of English and American physicians.

The second division of medical observers take the celebrated M. Louis of Paris as their exemplar, and attempt to subject all the important phenomena and results of disease to mathematical enumeration and comparison. By this class of observers we are told that in an hundred cases of typhoid fever, for example, certain rose-colored spots may be observed on the chest and abdomen in sixty of them, diarrhœa or thin fæcal discharges in seventy-five, delirium more or less in eighty, and so on in numerical ratio with each important symptom of the disease. To determine the value of remedial agents, a certain number of cases of disease, pneumonia for example, are selected, and perhaps one hundred of them subjected to venesection as a leading item in the treatment. The duration of the disease and the per centage of deaths are carefully noted. Another hundred cases are treated chiefly with antimonials, and the results noted in the same manner. The relative value of these remedial agents is then determined by a comparison of the results thus obtained. This is styled emphatically the *numerical* method of observation. And though it ranks among its followers some of the most renowned physicians of Paris, and some of well-deserved reputation in our own country, whose researches have indeed greatly enriched our professional literature; yet, as a method of therapeutical observation, it is inherently defective.

When any two or more sets of phenomena, or the effects of any two remedial agents, are to be compared with each other, it is necessary that each should present an absolute constancy or uniformity both in its own nature and the circumstances capable of influencing it. It requires but a limited knowledge of diseases to be satisfied that they present no such constancy. The special character of almost all forms of morbid action changes from day to day. An organized tissue, involved in inflammation, may present to-day simple increased vascularity with heat and pain, to-morrow be infiltrated with liquor sanguinis, and the day following the effused fluid be organized into false structure. And as all these changes necessarily modify more or less the effects of remedial agents, it is obvious that to make a *numerical* comparison, the results of which shall be free from error, two things are essential. First, all the cases embraced in the enumeration must present the same *grade* of morbid action, occurring in individuals of the same temperament and exposed to similar influences. Second, all the cases must be brought under treatment at the same relative stage of their progress. A moderate experience will satisfy all of you that these are conditions extremely difficult to fulfil in practice. Without their most rigid observance, however, the results of an application of the numerical method of observation to therapeutical investigation can be but little better than a series of errors. And yet M. Louis himself almost wholly disregarded these conditions. Thus, in twenty-six cases of one form of disease reported by him to determine the value of blood-letting in their treatment, the bleeding was practised in two on the *third* day; in one on the *fifth;* in four on the *sixth;* in one on the *eighth;* in two on the *ninth;* in four on the *tenth;* in two on the *fourteenth;* in two on the *sixteenth;* in four on the *seventeenth;* in one on the *twenty-second;* and in two on the *twenty-fifth.* No enlightened clinical observer will be surprised that so indiscriminate and unequal an application of so potent a remedial agent as blood-letting should have led M. Louis numerically to the conclusion that it possessed little or no power either to cut short or mitigate the disease under investigation. The same indiscriminate application of antimony, mercury, opium, quinine and alcoholic stimulants has been made by Louis and his followers in the treatment of pneumonia and continued fevers, and with very similar results. Thus one after another of our most powerful remedial agents, subjected to investigation by this method, have been either condemned or shown to possess but little actual control over the progress of disease. As a legitimate offspring of this numerical system, modern authors and essayists have filled our literature with statistical tables, embodying what purports to be the results of different methods of treatment in the same disease.

Thus after each epidemic of cholera, we have had statistical tables embracing several hundred cases, some treated chiefly by opiates, others by stimulants, and still others by nothing except cold water internally, with pounded ice and salt externally; and yet the per centage of deaths under each method of treatment was nearly the same. These therapeutical results, flowing directly from a system of observation conducted according to the forms, and presenting all the fascinating simplicity and precision of mathematical demonstrations, have exerted a great influence over the minds of the profession, and have contributed very largely to the establishment of that general distrust in the efficacy of remedial agents, and that disposition to follow an *expectant* system of medication even in the most acute diseases, which has been so rapidly on the increase in the profession during the last fifteen or twenty years. These same results have been chiefly instrumental in preparing the way for a revival of an ancient doctrine concerning the specific character of all the more important diseases; their tendency to run a definite course or period of time, regardless of therapeutical interference; and their amenability only to the all-controlling *powers* of *nature*—the *vis medicatrix natura* of the ancients. Fascinating as has been the numerical system of observation, introduced by the Parisian school of M. Louis, and extensive as has been its influence upon the profession, it is nevertheless based upon data entirely destitute of those qualities necessary to constitute elements in a mathematical comparison. Two or more objects to be compared by numerals must either be absolutely equal, or bear a certain mathematical ratio to each other. But what equality is there between two cases of disease, one in a previously robust and healthy individual, the other attacking its victim when already exhausted by over exercise, mental anxiety, or an insufficient supply of the necessaries of life? Or, what equality is there between three cases of fever, one in the third day of its progress, one in the fifteenth, and another in the twenty-fifth? And what uniformity of result could the physician expect who should use blood-letting or any other active remedial agent, alike in all such cases? Every enlightened physician knows that a remedy which might be eminently beneficial if applied to the treatment of an inflamed tissue at that early stage when the morbid process consisted in simple increased determination of blood to, and ts accumulation in the vessels of the part, might only hasten a fatal termination if used in the same manner at a later period when the tissue had become infiltrated with plastic lymph and the patient exhausted from the continuance of disease. And yet it is to such elements, absolutely variant in their nature, changeable from day to day, and consequently wholly incapable of being expressed in figures, that the numerical system of observation has been applied extensively during the last half century.

The application is as unphilosophical as its results are fallacious.

Another division of those who may be ranked in the school of observation have sought to advance our knowledge of the nature and tendencies of disease, by observing its effects as exhibited under the knife of the morbid anatomist and the microscope. The number included in this division is not large; neither have they succeeded in revealing to us the essential nature of a large class of diseases, but they have developed with much accuracy the tendencies and final results of morbid action, and thereby added a most interesting and important department of science under the title of microscopic and pathological anatomy. They might be appropriately styled the anatomical school of observers.

The second great class of medical investigators I have denominated the rational or theoretical.

The great object of this class has been to go a step or two beyond the mere observation and classification of facts. They have ever been inquiring after the why and the wherefore; or, in other words, endeavoring to invent some hypothesis which would explain the origin of the various facts ascertained by observation. The great leading object of this class has been to trace all morbid action to some primary starting point, or to discover a few fundamental principles which would explain all the more common and complex phenomena of disease.

Many have been the theories invented and ingenious the reasoning by which they have been sustained.

At one time the fluids of the body were regarded as furnishing the first link in the chain of morbid action, and all diseases were attributed to the processes of fermentation and concoction. At another time the doctrines of an exclusive solidism prevailed, and we had the long prevalent theories of *irritation* as modified by Brown, Darwin, Cullen, Broussais, and Rush. One of the latest and most comprehensive attempts at theorizing in medicine, was made by one of our own countrymen, and a resident in the Mississippi valley. I allude to the late Dr. Metcalf, who wrote a very voluminous and learned work to prove that *caloric* is not only the great motor power of the universe, but also the active or efficient agent in producing all vital phenomena. According to his doctrine, light, electricity, and magnetism, are only different manifestations of caloric; and to the influence of the latter he attributed alike the sublime movements of the planets, and the minutest molecular changes which take place in the human body in health and disease. It is from this class of theoretical investigators, that have emanated nearly all the special *pathys* and *isms* of the present and past generations. And yet theoretical speculations have not been altogether unfruitful as a means of advancing medical science. On the contrary, the most fanciful theories have often stimulated their authors and advocates to the collection of a much more extended series of facts, and to the performance of experiments which have resulted in the discovery of new facts and truths of great importance. And this leads us directly to the third and last method of inquiry, to which we shall call your attention, namely, that of direct *experiment*.

The theorist reasons from analogy, and speculates on mere probabilities. The simple observer collects, classifies, and practically applies such facts and phenomena as come voluntarily within his reach. But the experimenter goes beyond both, and by his own acts brings to light new phenomena for observation, and thereby makes positive additions to our stock of knowledge. Unlike both the other methods of investigation, the experimental is almost exclusively of modern origin. And yet to it are we indebted for a large proportion of the most important facts embodied in the several branches of medical science. To it also must we look as the most direct and efficient method of still further advancing the interests of our profession, and through it, the welfare of our race. But the passing hour will not permit a more extended review of these various methods of investigation.

As you have doubtless already anticipated, these methods have led their followers to the adoption of equally diverse and numerous therapeutical systems or methods of treating disease. These have been variously classified by different writers, but the arrangement proposed by Renouard is at once the most simple and comprehensive. He embraces all therapeutical systems under the three following heads, viz: the synthetical, the analytical, and the expectant. Under the first head are included all those methods of treatment which are founded on simple experience, without reference to the *modus operandi* of the medicines employed, or the particular elements which constitute the disease under treatment.

The synthetical system of Renouard is consequently nearly identical with that which has been denominated empirical by other writers. According to this system, it is sufficient to determine the particular disease under which the patient is laboring, and to apply such remedies as the accumulated experience of the past has shown to be most efficacious for its removal. It is by far the most ancient system, and even at the present day includes among its followers a very large proportion of the active practitioners of the healing art. It is the legitimate offspring of what I have styled the clinical method of observation.

The analytical method of therapeutics, instead of treating disease as a whole, or, to use the language of another, "entirely *en masse*," requires the practitioner to know something concerning both the *modus operandi* of his remedial agents and the several elementary morbid actions which constitute the disease. Thus, in a case of inflammation, the analytical therapeutist recognizes the elementary morbid processes of sanguineous determination, congestion and infiltration, with increased sensibility of structure, and directs his remedies with a view of removing these several elements independent of each other. This method has been styled by some rational or physiological; and so far as the present state of medical science will permit its adoption, it is far more satisfactory than the preceding or synthetical method.

The third and last therapeutic method to which I shall invite your attention is the *expectant.*

This method, as practised at the present day, is of very modern origin. The ancient school of clinical observers have been claimed as advocates of expectancy in medicine; but the claim is well founded only in part. Whatever approach to an expectant method of treatment discernible in the teachings of the ancients, is plainly founded on their ideas of concoction, of crises or critical days, and critical evacuations. Instead of being an absolute system of expectancy, or the withholding of active measures, it was rather a reservation of these, to be used only at such times as would favor the supposed crisis.

Modern expectancy is entirely different from this, and is easily traceable to three co-operating influences. First, the theoretical doctrines concerning the origin of all diseases from irritation, excitement and debility, reached the climax of their influence during the last half of the eighteenth century, and under the guidance of such men as Cullen and Rush, a bold and sometimes reckless use of the most active medicines became popular and almost universal. Disease was regarded much in the same light as an enemy to be attacked, subdued and removed from the system. And he who applied the most potent articles of the Materia Medica with the greatest boldness often attained the highest degree of popularity. The most active depletions upon the one hand, and the most diffusible stimuli upon the other, constituted the daily armory of the practitioner. Doctrines which inculcated measures of so heroic a character, could scarcely fail to engender excesses, that would sooner or later induce a reaction in the opposite direction, both in and out of the profession. Such being the condition of medical practice at the dawn of the nineteenth century, when the rapid advances in organic chemistry, the facts developed by direct experiments in physiology, and the researches of the morbid anatomists, were rapidly undermining all former theories of disease, revealing the composition of the solids and fluids of the system, and establishing more reliable means of diagnosis, it was both to be expected and desired that whatever was too bold or severe in these methods of practice, founded on former theories, should be corrected. But at this time a second influence arose in the form of a numerical method of observation as applied to therapeutics, and with its inherent fallacies rapidly developed a distrust in the efficacy of all remedies, as I have already explained. The third influence was almost a necessary result of the second, and consisted in the revival of the doctrine that nearly all important diseases are specific and self-limited in their character, and consequently incapable of being cut short by active medication. It is thus seen that the present expectant system of therapeutics is not so much a legitimate deduction from true clinical observation as it is the rebound from former systems of excessive medication, aided and directed by a false application of the numerical method of inquiry. According to this, now popular, system, diseases are not cured by medication, but by the "recuperative powers of nature." They are no longer enemies to be met and subdued, but simply unpleasant visitors, to be carefully watched and guided until they voluntarily take their leave.

To the advocates of this system I would respectfully propound a few serious inquiries. And first of all, what is this much talked of "*nature?*" And in what consists her wonderful "recuperative powers," on which we must rely for all really curative effects? If the proper office of the physician is to wait upon *nature* and *aid* her efforts, pray tell us what she is and how we may distinguish *her efforts* from the real effects of disease. I fear that fashionable words and popular phrases, are as often used to cover the ignorance of the writer as to convey definite ideas. But, second, what is meant by self-limited diseases, of which we hear so much in these days? That there are some which merit this title is readily admitted. Small-pox, for example, has its definite periods of incubation, development, maturity, and decline; so definite, indeed, that the physician can calculate the days, and almost the hours, with certainty. But who can tell us the self-constituted limits of typhoid fever, for example; or point us to the definite and uniform succession of its phenomena? Does it finish its natural course in two weeks or six? On what day of its progress do the intestinal symptoms appear, or the rose-colored spots upon the abdomen? Every unbiassed clinical observer knows that the disease has neither a definite limit to its duration, nor a fixed order of succession in its ordinary phenomena. And yet both the forms of continued fever, and most of the phlegmasia, are spoken of as self-limited diseases by the modern advocates of expectancy with as much complacency as though their limits were as well defined as the small-pox or measles. No reform is more needed at this day, than one which should insure greater precision and correctness in the use of language.

But, gentlemen, I must hasten these observations to a close. I have endeavored to call up before you in rapid review, the various methods of investigation with the therapeutic systems to which they have given rise, not for the purpose of condemning one and recommending another, but rather to glance at the advantages and defects of each, that you might in the further prosecution of your studies the more readily avail yourselves of the advantages of the former, and avoid the errors to which you might be conducted by the latter. Each of the systems of investigation to which I have alluded, if judiciously pursued, will aid in advancing the science and art which we are all desirous of cultivating. The method of *simple observation* must ever hold the first place; but to make its results valuable, the observations must be made with great care, minutely recorded, and only such placed in juxtaposition as are absolutely similar.

I have said that the observations must be *minutely recorded.* Here is one of the greatest failures in our literature. A very large share of the observations of physicians, if recorded at all, are done so in such general terms as to express much more nearly the mere *opinions* of the writer than the actual facts he has observed. Young gentlemen, if you would alike enhance your own knowledge and advance the sciences you cultivate, learn early to record accurately and minutely the important facts that come under your observation. Not only learn to observe carefully, and record accurately your observations, but classify them, reflect upon them, inquire freely the why and the wherefore. In other words *theorize*, gentlemen. Not, however, for the purpose of adding to the many discarded speculative systems of medicine, but for the legitimate and important purpose of ascertaining wherein the observed facts are defective, and how means or experiments can be devised, which, if carefully conducted, will elicit new facts to supply the deficiency. In this way reflection and theorizing may be made of great advantage to science. It is, indeed, when kept within proper limits, the appropriate link between the system of simple observation and that of experimental inquiry. On the latter you cannot bestow too much attention, for in no department is our American medical literature so defective as in the results of direct experimental investigations.

As we find use for all the great systems of investigation, so in the present state of our knowledge, must we sometimes resort in the treatment of diseases to all the therapeutical methods which have been described. There are some diseases, as periodical fevers, syphilis, etc., which are best treated "*en masse*," by remedies which partake of the character of specifics, and their treatment must consequently be included under the synthetic system. There are many other diseases, like the phlegmasia, whose elements are so well known that remedial agents can be chosen to counteract each in its turn, and their treatment must belong to the analytical or rational method. Again, there are other forms of disease, so obscure and hitherto so little under the control of any methods of treatment to which they have been subjected, that every physician is justified in adopting, in relation to them, a strictly expectant system of medication. Yet the great object of all our researches should be to so analyze the phenomena of all complex diseases, that their elements can be clearly comprehended, and remedial agents so chosen as to counteract each of these elements as they are presented to the observation of the physician at the bedside of the patient. This would bring all treatment under the analytical method, which is the only philosophical system of therapeutics.

Gentlemen, my task is done. But before I resume my seat, it will not be deemed out of place for me to allude to another subject.

Since we last met in these halls, two of those who have long been accustomed to occupy places by our side, and annually impart to the classes here assembled, the ample stores of knowledge which they had garnered from the fields of science, are missing from their places. I am happy, however, to state that neither of them have been removed by the grim messenger which we all so much dread.

One of them in the full vigor of life and health, and amply supplied with the good things of this world, has left the toils and cares of the physician for more congenial pursuits. The other, borne down by failing health, has been compelled to abandon at least temporarily the practice of a profession which he loved and honored, and leave a station which he had long filled with honor to himself and much usefulness to the alumni of our college. I need not say that we have parted with them with deep regrets, and that their former labors with us will be remembered and cherished so long as we continue to assemble from year to year in these halls. But while I allude to their absence with feelings of sadness, I am happy to introduce to you their successors, Professors Byford and Rauch, as men every way fitted to maintain the character of their positions, and we trust greatly advance the usefulness of the departments they have been called to teach. With these observations, gentlemen, I must again bid you welcome to these halls, and to all the advantages it is in the power of our faculty to afford you in the acquisition of useful knowledge.

